# RARE CASE OF CAROLI DISEASE WITH CHOLEDOCHODUODENAL FISTULA AND SUMP SYNDROME

**DOI:** 10.51894/001c.122815

**Published:** 2024-08-30

**Authors:** Pujyitha Mandiga, Rene Peleman

**Affiliations:** 1 Gastroenterology Ascension Macomb Oakland

## 10

### INTRODUCTION

Choledochoduodenostomy (CDDS) is a common procedure performed in patients with recurrent, impacted bile duct stones. We present a unique case of a patient with Caroli disease and repeat choledocholithiasis with CDDS presenting with a distal choledochoduodenal fistula (DCDF) and SUMP syndrome.

### CASE DESCRIPTION

A 76-year-old female with a past medical history of Caroli disease, multiple abdominal surgeries including CDDS, cholecystectomy secondary to recurrent choledocholithiasis presented to the clinic with abdominal pain. Further evaluation with an MRCP revealed filling defects in the distal common bile duct suggestive of choledocholithiasis. She underwent ERCP which revealed DCDF. Multiple small stones were extracted with purulent drainage with failed cannulation of the primary system. She developed severe right upper quadrant pain and nausea shortly after ERCP and presented to the emergency department. IV antibiotics were started due to concerns of cholangitis. IR placed a PTC. A repeat ERCP revealed old choledochoduodenostomy and internal and external biliary drain within the bile duct, and without cannulation of the intrahepatic duct. Fluid cultures grew *Enterococcus faecium* and antibiotics were added and discharged home. Two weeks later, a biliary catheter was removed two weeks later after gadolinium spillage of contrast into the bowel was seen, showing patency.

### DISCUSSION

Caroli disease is a congenital disorder presenting with large intrahepatic ductal dilation with an increased risk of hepatic fibrosis. There is bile stagnation leading to recurrent cholelithiasis. CDDS is a procedure performed in recurrent choledocholithiasis leading to gallstone pancreatitis and acute cholangitis despite cholecystectomy. CDDS creates a connection between the proximal common bile duct (CBD) and the 1st portion of the duodenum. The bile no longer drains via the distal CBD, creating a reservoir called a “sump”. Lithogenic bile, debris, or calculi accumulate causes recurrent cholangitis or choledocholithiasis. DCDF occurs at the entrance of the distal CBD into the 2nd part of the duodenum and is caused by impacted stones in 90% of the cases. DCDF is a rare complication from CDDS leading to SUMP syndrome. The impacted stone erodes the CBD wall leading fistulation into the duodenum. The diagnosis of DCDF is made with ERCP. Treatment requires fistulotomy, sphincterotomy, stone removal, and biliary drainage.

